# Genetic Variants of Interleukin-8 and Interleukin-16 and Their Association with Cervical Cancer Risk

**DOI:** 10.3390/life15020135

**Published:** 2025-01-21

**Authors:** Rafał Watrowski, Eva Schuster, Stefan Polterauer, Toon Van Gorp, Gerda Hofstetter, Michael B. Fischer, Sven Mahner, Robert Zeillinger, Eva Obermayr

**Affiliations:** 1 Department of Obstetrics and Gynecology, Helios Hospital Muellheim, Teaching Hospital of the University of Freiburg, Heliosweg 1, 79379 Muellheim, Germany; rafal.watrowski@gmx.at; 2 Faculty of Medicine, University of Freiburg, 79106 Freiburg, Germany; 3 Molecular Oncology Group, Department of Obstetrics and Gynecology, Comprehensive Cancer Center-Gynecologic Cancer Unit, Medical University of Vienna, Waehringer Guertel 18-20, 1090 Vienna, Austria; eva.schuster@meduniwien.ac.at (E.S.); stefan.polterauer@muv.ac.at (S.P.); robert.zeillinger@muv.ac.at (R.Z.); 4 Division of Gynecologic Oncology, University Hospital Leuven, 3000 Leuven, Belgium; toon.vangorp@uzleuven.be; 5 Leuven Cancer Institute, Catholic University of Leuven, 3000 Leuven, Belgium; 6 Department of Pathology, Medical University of Vienna, Waehringer Guertel 18-20, 1090 Vienna, Austria; gerda.hofstetter@meduniwien.ac.at; 7 Department of Blood Group Serology and Transfusion Medicine, Medical University of Vienna, Waehringer Guertel 18-20, 1090 Vienna, Austria; michael.b.fischer@meduniwien.ac.at; 8 Center for Biomedical Technology, Department for Biomedical Research, Danube University Krems, Dr.-Karl-Dorrek-Straße 30, 3500 Krems, Austria; 9 Department of Gynecology, University Medical Center Hamburg-Eppendorf, 20246 Hamburg, Germany; sven.mahner@med.uni-muenchen.de; 10 Department of Obstetrics and Gynecology, University Hospital, Ludwig-Maximilians-University Munich, 81377 Munich, Germany

**Keywords:** cervical cancer, gynecologic cancer, interleukin-8, IL-8, CXCL8, interleukin-16, IL-16, single nuclear polymorphism, chemokines, genetic variant

## Abstract

Background: Cervical cancer (CC) is the fourth most common cancer diagnosis in women worldwide. Infection with high-risk human papillomavirus (HPV) is a critical but not determinative condition for CC development, as several co-factors modulate the progression of HPV-associated cervical lesions. Interleukin-8 (IL-8) and Interleukin-16 (IL-16) are chemokine-like interleukins involved in the pathogenesis of various cancers. Singular studies in Asian populations have suggested a potential role of IL-8 rs4073 (−251 A>T) and IL-16 rs1131445 (3′UTR T>C) in cervical carcinogenesis. Methods: A case-control study was conducted in a European cohort of 339 women, including 126 CC patients and 213 controls. Four common IL-8 SNPs, rs4073 (−251 A>T), rs2227306 (+781 C>T), rs1126647 (+2767 A>T), and rs2227543 (+1633 C>T), and four IL-16 polymorphism, rs4778889 (−295 T>C), rs11556218 (3441 T>G), rs4072111 (1300 C>T), and rs1131445 (3′UTR T>C), were assessed using RFLP-PCR and analyzed under seven inheritance models. Subgroup analyses were stratified by menopausal status (age threshold 51 years), disease stage, and histological subtype. Results: IL-16 rs4072111 was significantly associated with an increased CC risk in premenopausal women in the co-dominant (*p* = 0.038), dominant (*p* = 0.022), and heterozygote (*p* = 0.045) models, identifying the T allele as the risk allele (OR 2.31, CI95% 1.17–4.56; *p* = 0.017). In women aged over 51, IL-16 rs4778889 was associated with CC in the heterozygote (*p* = 0.048) and overdominant (*p* = 0.042) models but not in the co-dominant model (*p* = 0.092). None of the analyzed SNPs significantly increased CC risk in the entire cohort. Specifically, neither IL-16 rs1131445 nor IL-8 rs4073, previously reported as risk factors in Asian populations, were associated with CC risk in this European cohort. Conclusions: These findings highlight the role of age stage in immunity and cancer susceptibility, suggest that IL-8 and IL-16 SNPs may function differently in cervical carcinogenesis compared with other cancers, and emphasize the importance of ethnic background in cancer risk, warranting further research.

## 1. Introduction

Cervical cancer (CC) is the fourth most diagnosed cancer and the fourth leading cause of cancer-related death among women worldwide, following breast, colorectal, and lung cancers [1]. The lifetime risk of developing CC is approximately 1.5%, with an estimated 660,000 new cases and 350,000 deaths estimated globally in 2022. CC accounts for 6.8% of all female cancer cases and 8.1% of female cancer deaths [1]. Despite advances in early detection and prevention, the mortality-to-incidence ratio remains high at around 53% [1]. Histologically, 75–85% of CC cases are squamous carcinomas, 12–13% are adenocarcinomas, and 3–5% are adenosquamous carcinomas [2,3]. The remaining 5% include rare subtypes, such as neuroendocrine, clear cell, serous carcinomas, lymphomas, and sarcomas [3,4]. Surgery with stage-adapted radicality is the first-line option for early-stage CC, while systemic treatments (radiochemotherapy, chemotherapy) are the first-line options for advanced stages [5,6]. The overall 5-year survival rate decreases significantly with advanced FIGO stages: 88% for stage I, 82% for stage II, 74% for stage III, and 12% for stage IV. Survival also declines with patient age: 84% for those under 40, 80% for ages 40–65, and 37% for those over 65 [7].

CC is an infection-related cancer, as the human papillomavirus (HPV) infection is a necessary, but not sufficient, cause of the disease [1,5,8]. HPV is detected in 96% of CC cases, yet most HPV infections resolve spontaneously within 8–16 months [8,9,10]. During the ages of greatest sexual activity, the prevalence of subclinical HPV infections in women can reach 40%, with an annual infection rate of 10–15%. In women over 30, the prevalence drops to 5–10% [8]. While the incidence of new infections decreases with age, the persistence of existing infections increases [8]. Among 448 known HPV types, 25 are classified as high-risk HPV (hrHPV), including HPV16, HPV18, HPV31, HPV33, and HPV45 [9]. HPV16 and HPV18 alone are responsible for approximately 70% of cervical cancers [9,10]. The prevalence of hrHPV infection peaks twice: first between the ages of 15 and 26 and again around 45 years [10]. These patterns correspond to two peaks in CC incidence: the first between ages 30 and 45 and the second between 60 and 80 years [11,12].

Although most hrHPV infections are transient, around 10% persist and can progress to low-grade squamous intraepithelial lesions (LSILs), high-grade squamous intraepithelial lesions (HSILs), and eventually invasive CC. Infections with HPV16 and HPV33 carry a markedly increased risk (~25-fold) of progressing to preinvasive or invasive cancer, whereas HPV16 and HPV31 have the lowest likelihood of spontaneous clearance [13]. LSILs regress spontaneously in about 60% of cases, while HSILs have a lower regression rate of around 25%; up to 18% of HSILs progress to invasive CC if untreated [14].

The variability in HPV infection outcomes and CC progression arises from a complex interplay of viral and host factors. On the viral side, HPV genotype, viral genetic and epigenetic modifications, and viral load are critical determinants of disease progression [15]. The host immune system, both innate and adaptive, is crucial in determining whether HPV infections are cleared, persist, or progress to cancer [16]. Host-related co-factors primarily are related to sexual and reproductive behavior, e.g., multiple sexual partners, early sexual debut, long-term use of oral contraceptives, high parity, cervicovaginal microbiome alterations, sexually transmitted co-infections (e.g., HIV and *Chlamydia trachomatis*), and further including lifestyle factors such as smoking [9,10,15]. Immunological changes have been documented in premalignant and malignant cervical lesions, including alterations in cytokine expression by cervical epithelial cells and by infiltrating leukocytes. These immune factors are detectable in biopsies from patients with HPV infections and different stages of CIN or CC. Additionally, leukocytes in the tumor microenvironment release signaling molecules that modulate processes like angiogenesis, chemotaxis, and apoptosis [16,17].

Family aggregation studies and heritability estimates indicate a substantial genetic component to CC susceptibility [15]. A genome-wide association study (GWAS) estimated that 24% of the variation in CC risk is due to common autosomal single nucleotide polymorphisms (SNPs), slightly lower than the 27% heritability estimate from family studies. Genetic variants associated with CC development identified in GWAS include lymphotoxin alpha (LTA), tumor necrosis factor (TNF), Paired Box 8 (PAX8), Cleft Lip and Palate Transmembrane Protein 1-like (CLPTM1L), and Human Leukocyte Antigen (HLA) genes, indicating a potential disruption in apoptotic and immune function pathways [15,18]. However, variants identified by GWAS explained only 2.1% of phenotypic variance, implying that a significant proportion of heritability is tagged by common SNPs with small individual effects [18].

Interleukins, a class of cytokines originally named for their role in leukocyte communication, are now recognized to be produced by a wide range of cells and play various roles in carcinogenesis. Numerous candidate gene studies have shown that SNPs in interleukin genes, such as IL-1, IL-6, IL-10, and IL-12, are associated with altered risks of HSIL and CC [19,20,21,22]. Among 41 known interleukins, IL-8 and IL-16 are often classified together as “chemokine-like interleukins” for their role in attracting immune cells to inflammation sites [23]. IL-8, also known as CXCL8 (C-X-C motif chemokine ligand 8), is a pro-inflammatory and pro-angiogenic chemokine within the CXC chemokine superfamily [24]. It is produced by neutrophils, monocytes, fibroblasts, endothelial cells, smooth muscle cells, and epithelial cells [24,25]. High IL-8 expression and its receptor activation are observed in multiple tumor microenvironment components, including cancer cells, endothelial cells, and tumor-associated macrophages, promoting angiogenesis, cell proliferation, survival, and migration [24].

The *IL8* gene, located on chromosome 4q12-q135, comprises four exons and three introns, encoding a 99-amino acid precursor protein processed into active isoforms. Its transcription is primarily regulated by NF-κB through TNF and TRAF6 pathways [24,26]. Polymorphisms in *IL8* can affect gene expression or protein structure, altering binding affinities and downstream signaling [26]. The most studied IL-8 SNPs are rs4073 (−251 A>T) in the promoter, rs2227306 (+781 C>T) in intron 1, rs2227543 (+1633 C>T) in intron 3, and rs1126647 (+2767 A>T) in the 3′UTR [26,27]. The rs4073 T allele is linked to higher IL-8 expression, with two to five times greater transcriptional activity than the A allele [28]. This SNP correlates with elevated IL-8 plasma levels, showing the highest expression in AA, intermediate in AT, and lowest in TT genotypes [29]. Rs2227306 in intron 1 also affects transcription and regulation [30]. Beyond oncology, *IL8* SNPs, especially rs4073, are linked to, e.g., asthma [30], acute coronary syndrome [29], and age-related macular degeneration [31]. In oncology, IL-8 SNPs are associated with glioma [32], osteosarcoma [33], and gastric [28], lung [34], hepatocellular [35], and nasopharyngeal cancers [36]. For the female genital tract, one study linked the TT genotypes of rs2227306 and rs1126647 to increased ovarian cancer (OC) risk [37]. In our previous study, three of the four SNPs (rs4073, rs2227306, and rs2227543) were associated with OC risk in postmenopausal but not premenopausal women. We also found that rs1126647 genotypes containing the T-allele were significantly linked to endometriosis-related OC subtypes, with TT homozygotes more frequent in these subtypes than in other OC subtypes (39% vs. 19%) [38].

IL-16, first identified in 1982 as a “lymphocyte chemoattractant factor” for CD4+ cells, is a multifunctional protein involved in immune regulation, cell migration, and cell cycle control [39,40]. It is produced by T lymphocytes, macrophages, dendritic cells, fibroblasts, mast cells, B cells, and bronchial epithelial cells [40,41,42]. While CD4 is its primary receptor, IL-16 can also bind to CD9 or function via CD4/CD9-independent mechanisms [43,44]. The *IL16* gene on chromosome 15q26.3 has seven exons and six introns and encodes two precursor isoforms produced by alternative splicing: a 636-amino acid Pro-IL-16 in immune cells and a 1244-amino acid neuronal variant (nPro-IL-16) [39,40,42]. Caspase-3 cleaves both into mature IL-16, a 121-amino acid protein with chemotactic and growth factor activities [41,42,43]. Both precursor forms migrate to the nucleus, where they act as nuclear transcriptional repressors [41,44,45]. IL-16 is linked to inflammatory and autoimmune diseases, such as asthma, multiple sclerosis, systemic lupus erythematosus (SLE), and rheumatoid arthritis (RA) [42,46,47,48], as well as inflammation-driven conditions like susceptibility to viral infections, depression, and cardiovascular diseases [42,49,50]. In cancer, IL-16 attracts CD4+ cells, showing both tumor-promoting and suppressive effects. Its role varies: in cutaneous T-cell lymphoma (CTCL), a pro-IL-16 mutation reduces p27KIP1, enhancing cell growth; in multiple myeloma (MM), IL-16 overexpression drives plasma cell proliferation; and in breast cancer, it recruits pro-tumor macrophages [43,50].

The most studied IL16 variants are rs4778889, rs11556218, rs4072111, and rs1131445 [50]. The rs11556218 (T>G) missense mutation in exon 6 is linked to higher IL-16 levels in TG/GG genotypes [51,52], with the G allele increasing the risk of lung [53], oral [54], nasopharyngeal [52], gastric [50,55,56], and colorectal cancer [55], as well as osteosarcoma [51], endometriosis [57,58], cardiovascular disease [50], and SLE [59]. Another missense mutation, rs4072111 (C>T) in exon 6, is associated with an increased risk of Parkinson’s [60], Alzheimer’s [61], and SLE [59], though its link to endometriosis [57,58] and gastric cancer [55,62] remains inconsistent. Rs4778889 (T>C), located in the promoter region, reduces promoter activity, with the T allele increasing asthma risk [46], while the C allele (TC/CC genotypes) is linked to a higher risk of renal cell carcinoma [63], gastric cancer [50], nasopharyngeal cancer [52], endometriosis [64,65], and SLE [59]. Rs1131445 (T>C) in the 3′-untranslated region (3′UTR) affects the miR-135b binding site, disrupting miRNA suppression and upregulating IL-16 expression [48,66]. The C allele is associated with an increased risk of colorectal cancer [67], RA [48], and SLE [48]. In gynecology, *IL16* genetic variants play a role in ovarian carcinogenesis. Our recent study found a strong association between OC risk and rs11556218 (G vs. T allele: OR 2.76, *p* < 0.0001) across all age groups, as well as with rs4778889 (C vs. T allele: OR 1.94, *p* = 0.016) in premenopausal women [68].

For CC, only one IL-8 SNP (rs4073) [69] and one IL-16 SNP (rs1131445) [66] have been studied, each in a single study, both limited to Chinese populations. For IL-8 rs4073, T-allele-containing genotypes (AT and TT) were significantly associated with CC, with TT homozygotes showing an increased risk of lymph node metastasis [69]. In the seminal study by Mi et al., rs1131445 in the miR-135b binding site of IL-16 3′-UTR was found to affect IL-16 protein expression by interfering with miR135b suppressive function and was significantly associated with the risk of CC. Patients carrying the rs1131445 C allele had higher serum IL-16 levels compared with non-carriers [66].

The remaining IL-8 SNPs (rs2227306, rs2227543, rs1126647) and IL-16 SNPs (rs4778889, rs11556218, rs4072111) have not been studied in CC. To fill this gap, we evaluated the association of four common IL-8 and four IL-16 polymorphisms with CC risk in a geographically and ethnically well-defined European cohort, including subgroup analyses by estimated menopausal status, histological subtype, and FIGO stage.

## 2. Materials and Methods

### 2.1. Study Design and Participant Characteristics

This case-control study evaluated four IL-8 SNPs, rs4073 (−251 A>T), rs2227306 (+781 C>T), rs1126647 (+2767 A>T), and rs2227543 (+1633 C>T), along with four IL-16 SNPs: rs4778889 (−295 T>C), rs11556218 (3441 T>G), rs4072111 (1300 C>T), and rs1131445 (3′UTR T>C). The analysis included blood samples from 339 Central European women, comprising 126 CC cases and 213 healthy controls. SNP genotyping was performed using the restriction fragment length polymorphism (PCR-RFLP) method.

The blood samples were sourced from the Molecular Oncology Group’s blood bank at the Medical University of Vienna, the coordinating center of a European biobanking project approved by the Ethics Committee of the Medical University of Vienna (EK-366/2003 and EK 1966/2020) and registered on ClinicalTrials.gov (NCT01763125). Blood samples for this study were selected from a collection obtained between 1996 and 2021, involving patients and controls recruited at the Medical University of Vienna and partner institutions across Europe. The samples used in the current analysis originated specifically from Austria, Poland, Germany, and Belgium. Women with metastatic cervical lesions and those with induced menopause (e.g., surgical or pharmacological) were excluded from the study. Menopausal status was estimated using an age cutoff of 51 years, reflecting the median age of menopause in Central Europe [70], and staging was adjusted to the revised 2018 FIGO classification [71]. Ethical compliance with the 1964 Helsinki Declaration and its amendments was ensured. Written informed consent was obtained from all participants, and data were anonymized and processed according to good scientific practice.

### 2.2. DNA Extraction and Genotyping

Peripheral blood was collected from all participants in EDTA tubes. Genomic DNA was isolated from white blood cells using the QIAamp DNA Blood Mini Kit (QIAGEN, Hilden, Germany) and then quantified using the QuantiFluor^®^ dsDNA System (Promega, Alcobendas, Madrid, Spain) and the QuBit Fluorometer (Thermo Fisher Scientific, Waltham, MA, USA). The single nucleotide variants of IL-8 and IL-16 were determined by analyzing fragment length polymorphisms of the respective PCR products (PCR-RFLP). Details on primers, annealing temperatures, and restriction enzymes are provided in Table 1. The amplicons were generated from 25 ng genomic DNA as a template in a 25 µL reaction mix, containing 5 pmol of the respective forward and reverse primers and MangoMix™ (Bioline, London, UK) providing MangoTaq™ DNA polymerase, MgCl_2_, and dNTPs. Amplification began with a hot start at 95 °C for 5 min, followed by 45 cycles of 30 s denaturation at 95 °C, 30 s annealing at specific temperatures (Table 1), and 60 s extension at 72 °C. A final extension was performed at 72 °C for 7 min. PCR products were digested with the respective restriction endonuclease (New England Biolabs, Ipswich, MA, USA) under the conditions given in Table 1. The restriction fragments were separated with capillary electrophoresis using the Fragment Analyzer™ Automated CE System (Advanced Analytical, Ankeny, IA, USA) and the DNF-905 dsDNA Kit (Agilent, Santa Clara, CA, USA). The sizes of the fragments were assessed using the software PROSize^®^ 3.0 version 3.0.1.6 (Advanced Analytical Technologies, Orangeburg, NY, USA).

### 2.3. Statistical Analysis

Genotype and allele frequencies were compared between cases and controls using the Chi-squared test, with Yates correction applied when cell counts were below five. Odds ratios (ORs) with 95% confidence intervals (CIs) and Fisher’s exact test were used to assess the effect of each SNP on CC risk. In the control group, Hardy–Weinberg equilibrium (HWE) was verified for all SNPs using the goodness-of-fit χ^2^ test. Age differences were analyzed with the Mann–Whitney test, considering a two-sided *p*-value ≤ 0.05 as statistically significant. Associations between SNPs and CC risk were evaluated using seven inheritance models (Table 2) [72,73]. All statistical calculations were conducted using the JASP statistical software v.19.0.0 for Windows [74] and the VassarStats Website for Statistical Computation [75].

## 3. Results

**Figure A1:**
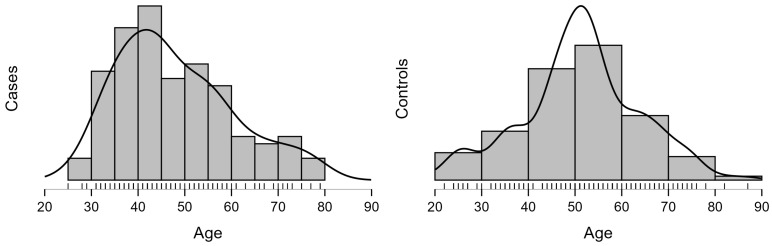
Age distribution and density between CC cases (at first diagnosis) and controls.

Within the cohort of 339 women, 126 were diagnosed with CC, and 213 were healthy controls. The median age of the cases was 45.5 years (range: 25–79), while the median age of the controls was 51 years (range: 18–87). The proportion of premenopausal patients, defined as those younger than 51 years, was higher among cases (61%) than controls (46%; *p* = 0.006), as shown in Table 3. As shown in Figure A1 (Appendix A), the disease peaked between the ages of 35 and 40, followed by the 40–45 age group. After menopause, most patients were diagnosed between 50 and 55 years, followed by the 55–60 age group.

As presented in Table 3, 50 out of 126 CC cases (40%) were diagnosed at an early FIGO stage (I-IIa), while 73/126 (58%) were diagnosed at advanced stages (IIb-IV). The most common histological type was squamous carcinoma, observed in 80% of patients (101/126), while 18% had non-squamous subtypes (adenocarcinoma or adenosquamous carcinoma). A detailed listing of histological subtypes and grades, as well as stratification by FIGO stage, is provided in Table A1.

The minor allele frequencies (MAFs) in the control group are presented in Table 4. As expected from other sources, the MAFs for IL-8 were typically high, with 46.7% for rs4073 (A allele), 43.7% for rs2227306 (T allele), 42.7% for rs2227543 (T allele), and 41.1% for rs1126647 (T allele). For IL-16 SNPs, the MAFs were 9.2% for rs11556218 (G allele), 13.6% for rs4778889 (C allele), 10.3% for rs4072111 (T allele), and 32.4% for rs1131445 (C allele). All MAFs in the control group were representative of the European population, as demonstrated by comparison with those reported in the gnomAD (Genome Aggregation Database) [76] and dbGaP Allele Frequency Aggregator (ALFA) databases [77]. The genotype distribution did not deviate from Hardy–Weinberg equilibrium in any case (see Table 4).

As shown in Table 5 and Table 6, in this ethnically homogeneous European cohort, no general association was observed between the IL-8 and IL-16 SNPs and the risk of CC. Specifically, no association was found for the G allele of rs11556218, which has been linked to many oncological and non-oncological conditions, nor for rs1131445 (T>C), the only IL-16 SNP previously investigated in relation to CC [66]. Additionally, in contrast to a solitary prior study investigating the IL-8 SNP rs4073 in CC [69], neither the genotype nor the allelic frequencies differed significantly between the CC group and the healthy controls.

A significant association, however, was observed between IL-16 SNP rs4072111 (C>T) and CC risk in premenopausal women. The association was especially strong (χ^2^ = 6.1) and significant (*p* = 0.014) in the allelic comparison, revealing the common C allele as protective and the minor T allele as a risk allele, with a corresponding OR of 2.31 (95% CI 1.17–4.56). Similarly, in the genotype analysis, being homozygous for the common CC genotype was associated with a significantly reduced CC risk compared with heterozygotes (CT).

Furthermore, regarding menopausal status, a weak association between IL-16 rs4778889 (T>C) and CC risk was noted for postmenopausal women in both the heterozygote (*p* = 0.048) and overdominant (*p* = 0.042) models, with the minor C allele being the risk allele. However, this association diminished below the significance level in the co-dominant model (*p* = 0.091) and allele frequency comparison (*p* = 0.164). These results are presented in Table 7.

No further associations were found when broken down by the estimated menopausal status for IL-16 rs11556218 (T>G) and IL-16 rs1131445.

Notably, none of the studied SNPs showed any association with histological type (squamous vs. non-squamous) or stage of disease—early vs. advanced—at first diagnosis.

## 4. Discussion

Persistent hrHPV infections initiate carcinogenesis primarily through the expression of the viral oncogenes E6 and E7, which inactivate the tumor suppressor proteins p53 and retinoblastoma protein (pRB), disrupt cell cycle control and activate telomerase, leading to cellular immortalization. E6 and E7 are implicated in every stage of cervical carcinogenesis and contribute to the evasion of host immune responses, interference with key signaling pathways like MAPK and mTOR, and reprogramming of the host cellular environment [9,78]. The tumor microenvironment is critical in regulating tumor progression, angiogenesis, and metastasis. This environment includes tumor cells, endothelial cells, cancer-associated fibroblasts, and infiltrating inflammatory cells controlling local cytokine networks. Among these, IL-8 (CXCL8) is a chemokine that activates intracellular signaling, mediating pro-tumorigenic effects such as epithelial-mesenchymal transition, survival, proliferation, migration, invasion, angiogenesis, and resistance to apoptosis [79]. IL-8 mRNA and protein are upregulated in CC tissues and cell lines compared with normal cervical tissues and are associated with the proliferation and migration of cervical epithelial cell lines [80,81]. A recent study confirmed that CXCL1, CXCL2, CXCL3, and CXCL8 (IL-8) are regulated by HPV16 and HPV18 E6/E7 and are overexpressed in CC biopsies, with higher expression associated with worse survival [82]. IL-8 levels in liquid-based cervical samples increase with the progression of intraepithelial lesions from low grade to high grade [83]. Similarly, IL-6 and IL-8 levels measured in cervicovaginal washings are higher in patients with invasive CC than in those with cervical intraepithelial neoplasia [84]. In addition, IL-6 and IL-8 show greater expression in the LSIL and HSIL groups compared with normal controls, with melatonin modulating both cytokines’ effect on the progression of neoplastic lesions in HPV infection [85]. In addition to its immune functions, IL-8 is a pro-angiogenic factor that regulates vascular endothelial growth factor (VEGF), matrix metalloproteinases, and other mediators through autocrine and paracrine pathways [86]. IL-8 also enhances the angiogenic capability of CC cells via G protein-coupled lysophosphatidic acid (LPA) receptors 2 and 3, Gi-mediated PI3K-Akt, PKC pathways, and NF-kB activation [87]. In a xenograft model, IL-8 promoted tumor growth and metastasis in vivo, while IL-8 antibody treatment reduced tumor volume, decreased lymph node metastasis, and improved animal survival. Thus, IL-8 blockade shows promise as an alternative approach for CC treatment [88]. Several studies confirmed that IL-8 protein expression correlates with clinical stage, distant metastasis, histological type, and histological grade of CC and that high IL-8 expression is linked to shorter survival in CC patients [79,80,89,90].

Given the diverse role of IL-8 in cervical carcinogenesis and the documented impact of genetic variants in other pro-inflammatory and pro-angiogenic cytokine genes [19,20,21,91], we expected that common SNPs in the IL-8 gene might alter CC risk. In a Chinese study, the T-allele-containing genotypes (AT and TT) of IL-8 rs4073 (−251 A>T) were significantly more frequent in CC patients compared with controls, and the TT genotype was associated with an increased risk of lymph node metastasis (OR = 2.917, *p* = 0.035) [69]. However, in our European cohort, no association was found between any of the IL-8 SNPs and CC risk. This lack of association appears robust despite the relatively small sample size, given the high MAFs of the IL-8 SNPs (ranging from 41% to 47%). Common variants with high MAFs are less prone to false positives than rare variants, especially in studies with modest sample sizes, as they provide more statistical power for detecting associations [92,93]. Thus, the absence of significant associations suggests that these variants likely do not play a major role in cervical carcinogenesis.

Furthermore, differences in the prevalence and impact of genetic variants with small effects between geographically/ethnically distinct populations, particularly between Asian, African, and European populations, are well documented [94,95] and have also been reported in relation to CC [96]. Variations in allele frequencies can be attributed to population-specific genetic drift, natural selection, and historical demographic events [97], whereas the functional significance of certain immune-related genetic variants may arise from transcriptome and epigenetic alterations influenced regionally by environmental and lifestyle factors, as well as by exposure to pathogens and toxins. These influences are reflected in changes to immune cell function and disease susceptibility [98,99].

The role of IL-16 in relation to CC is less well researched as compared to IL-8. IL-16 SNPs have been implicated in several cancers [50], with the G allele of rs11556218 associated with an increased risk of gastric [50], lung [53], oral [54], and ovarian [68] cancers. However, little is known about the role of IL-16 in cervical HPV infection and CC, particularly regarding the influence of IL-16 genetic variants. A Chinese study demonstrated that rs1131445, located in the miR-135b binding site of the IL-16 3′-UTR, affects IL-16 protein expression by interfering with the suppressive function of miR-135b [66]. MicroRNAs are small (18–30 nucleotides) non-coding RNAs regulating the function of other genes primarily by “inhibiting the production of protein from mRNAs to which the microRNAs can bind by base pairing” [100]. As negative regulators of gene expression, they play a fundamental role in cell development and carcinogenesis (the discovery of micro RNAs has been awarded the 2024 Nobel Prize) [100]. Patients carrying the rs1131445 C allele have higher serum IL-16 levels than non-carriers. This interference is significantly associated with an increased risk of cervical cancer in Asian patients [66]. In contrast, in our European cohort, no association between rs1131445 and CC risk could be observed.

We are the first to report associations between two IL-16 SNPs, rs4072111 (C>T) and rs4778889 (T>C), when stratified by menopausal status. In premenopausal women, the common C allele is imposed as protective and the minor T allele as a risk allele regarding rs4072111 (C>T) and CC. In contrast, in postmenopausal women, we observed an association between IL-16 rs4778889 (T>C) and CC for individuals carrying the minor C allele. However, the latter observation was limited to heterozygote and overdominant models.

This result emphasizes menopause as a critical threshold in local and systemic immunological processes, including those relevant to HPV-associated carcinogenesis. Our previous studies clearly showed the different impacts of IL-8 and IL-16 SNPs depending on menopausal status [38,68]. Epidemiological data demonstrate a bimodal distribution of CC, with peaks occurring between 30–39 years of age and 60–69 years of age [12]. In the present study, the majority of CC cases were diagnosed as premenopausal, but 40% occurred in postmenopausal women. This bimodal distribution may reflect different peaks in HPV acquisition [11], as well as variations in epigenetic and environmental factors influencing CC development. Elderly-onset CC patients exhibit a significantly higher frequency of NOTCH1 and TP53 driver mutations compared with younger patients, along with a notably higher tumor mutational burden [101]. Additionally, patients aged 65 and older with squamous CC show a higher frequency of PIK3CA mutations, which are associated with increased mutation rates in other genes involved in key cancer-associated pathways, such as tyrosine kinase receptors, K-Ras/BRAF/MAPK and the Wnt/β-catenin pathway [102]. However, as the functional consequences of the rs4778889 genetic variants on IL-16 protein expression in CC are not known, and the association in our study was limited to two inheritance models in postmenopausal women, further investigation is warranted.

A stronger and more definitive association was observed, in contrast, in premenopausal women, where the common C allele was imposed as protective and the minor T allele as a risk allele of rs4072111 (C>T) regarding CC risk. This observation is particularly intriguing, as nPro-IL-16 (the larger isoform of the IL-16 precursor) was originally believed to be exclusively expressed in hippocampal and cerebellar neurons [45]. Similar to Pro-IL-16 in immune cells, nPro-IL-16 is cleaved by caspase-3 to release mature IL-16 [45]. In the nervous system, nPro-IL-16 plays a role in upregulating the transcription factor c-fos, promoting neurite outgrowth, and interacting with neurotransmitter receptors and neuronal ion channels [45]. Rs4072111 (C>T) is a missense mutation in exon 6, leading to a proline-to-serine substitution (Pro434Ser) in nPro-IL-16 [57,58]. The T allele has been linked to an increased risk of Parkinson’s disease [60] and Alzheimer’s disease [61]. More recently, nPro-IL-16 has also been detected in extraneuronal tissues, such as arthritic cartilage [103], indicating that its role beyond the nervous system is still not fully understood. Additionally, rs4072111 has been associated with gastric cancer [54,62] and endometriosis [57,58], with the T allele linked to severe endometriosis stages [57].

Our results suggesting a potential association between rs4072111 and CC are therefore noteworthy. The cervix and parametrium contain nerve fibers [6,104], and perineural invasion (PNI) is a critical factor in CC progression, establishing pathways for CC colonization and metastasis along nerves [105,106]. Molecules initially identified in nerve tissues are now recognized for their roles in tumor-nerve interactions, influencing PNI and directly affecting CC cell growth. For instance, neurotrophins like nerve growth factor (NGF) and brain-derived neurotrophic factor (BDNF) are overexpressed in CC cells and promote PNI through interactions with p75 and Trk receptors [105,107]. NGF induces CC cell proliferation and migration [108], and high levels of NGF and TrkA are associated with PNI in early-stage cervical cancer [109]. The neuropeptide neuromedin B (NMB), also produced by CC cells, induces PNI by reprogramming Schwann cells, driving morphological and transcriptional changes, promoting proliferation and migration, and initiating PNI via CCL2 secretion and stimulation of axon regeneration [110]. It can be hypothesized that nPro-IL-16, through its role in nerve growth and differentiation, may contribute to CC expansion similarly to neurotrophins. Additionally, the secreted form of IL-16, produced from nPro-IL-16 cleavage, could further influence cervical carcinogenesis. Therefore, the potential role of rs4072111 (C>T), including its impact on the extracellular functions of IL-16, warrants further investigation.

To conclude, our study has several strengths. First, it is the first to investigate IL-8 and IL-16 genetic variants and their impact on CC risk in a European population. Second, the differentiated approach, which considered menopausal status, revealed that some associations were specific to premenopausal or postmenopausal women. Third, the observed association of rs4072111 with CC may encourage further research into the role of IL-16 and its neuronal precursor molecule in carcinogenesis.

However, our study also has limitations. The moderate sample size may have reduced the statistical power for SNPs with low MAF, particularly for IL-16. Additionally, as we did not have data on the expression levels of IL-8 and IL-16 in tissues or peripheral blood, our results, while assessing risk, do not allow us to determine the possible mechanisms underlying the observed or potentially missed associations. Third, we used an age threshold of <51/≥51 years as a proxy for menopausal status, a recommended approach when direct information on menstrual history is unavailable [111,112]. However, our data did not allow for distinguishing the effects of ovarian function cessation (menopause) from age-related processes, such as the progressive accumulation of DNA damage, reduced DNA repair capacity, increased cellular senescence, and accumulating epigenetic alterations [99,113]. Additionally, lifestyle factors (e.g., diet, physical activity, circadian disruption) and chronic low-grade systemic inflammation (“inflammaging”) further exacerbate age-related vulnerabilities to malignancies [114,115]. Thus, our findings that SNPs with low penetrance can impact CC risk differently in younger and older women may reflect influences of menopausal status, age, or their combined effects [99].

## 5. Conclusions

This study is the first to report a significant association between IL-16 rs4072111 and increased CC risk in women aged under 51 and between IL-16 rs4778889 and CC risk in women aged over 51. None of the eight analyzed SNPs significantly affected CC risk in the entire cohort. Specifically, neither IL-16 rs1131445 nor IL-8 rs4073, previously linked to CC risk in Asian populations, were linked to CC risk in this ethnically homogenous European cohort. Our results highlight the role of age-stratified analysis in cancer susceptibility and underscore the importance of ethnic background in assessing genetic risk factors. Additionally, they suggest that IL-8 and IL-16 SNPs may influence CC risk differently than other cancers, likely due to the specific relationship of CC carcinogenesis with HPV.

## Figures and Tables

**Table 1:** PCR-RFLP of IL-8 and IL-16 SNPs. Primers for amplification, annealing temperature, and restriction enzyme for digestion.

SNP	Primer Sequence	Annealing Temperature	Digestion (Enzyme, Temperature, Duration)	Fragment Size (bp)
**IL-8**				
rs4073 (T>A)	Forward: 5′-TCATCCATGATCTTGTTCTAA-3′ Reverse: 5′-GGAAAACGCTGTAGGTCAGA-3′	55 °C	Mfe I, 37 °C, 25 min	T/T: 524 A/A: 449 + 75
rs2227306 (C>T)	Forward: 5′-CTCTAACTCTTTATATAAGGAATT-3′ Reverse: 5′-GATTGATTTTATCAACAGGCA-3′	50 °C	EcoR I, 37 °C, 25 min	T/T: 203 C/C: 184 + 19
rs2227543 (C>T)	Forward: 5′-CTGATGGAAGAGAGCTCTGT-3′ Reverse: 5′-TGTTAGAAATGCTCTATATTCTC-3′	55 °C	NIa III, 55 °C, 35 min	T/T: 397 C/C: 234 + 163
rs1126647 (A>T)	Forward: 5′-CCAGTTAAATTTTCATTTCAGGTA-3′ Reverse: 5′-CAACCAGCAAGAAATTACTAA-3′	50 °C	BstZ17I, 37 °C, 25 min	A/A: 222 T/T: 198 + 24
**IL-16**				
rs4778889 (T>C)	Forward: 5′-CTCCACACTCAAAGCCTTTTGTTCCTATGA-3′ Reverse: 5′-CCATGTCAAAACGGTAGCCTCAAGC-3′	60 °C	Ahd I, 37 °C, 25 min	T/T: 280 C/C: 246 + 34
rs4072111 (C>T)	Forward: 5′-CACTGTGATCCCGGTCCAGTC-3′ Reverse: 5′-TTCAGGTACAAACCCAGCCAGC-3′	67 °C	BsmA I, 55 °C, 35 min	C/C: 164 T/T: 140 + 24
rs11556218 (T>G)	Forward: 5′-GCTCAGGTTCACAGAGTGTTTCCATA-3′ Reverse: 5′-TGTGACAATCACAGCTTGCCTG-3′	60 °C	Nde I, 37 °C, 25 min	G/G: 171 T/T: 147 + 24
rs1131445 (T>C)	Forward: 5′-GAGATCATTCACTCATACATCTGG-3′ Reverse: 5′-TCATATACACGCTGGTTCCTTCTG-3′	62 °C	BsaA I, 37 °C, 25 min	T/T: 460 C/C: 300 + 160

rs number (Ref-SNP)—ID for each SNP assigned by dbSNP.

**Table 2:** Genetic models studied.

Model	Definition	Interpretation
Co-dominant (=General Test of Association)	AA vs. Aa vs. aa	Compares the impact of each genotype (AA, Aa, aa) on the outcome (all three genotypes are compared simultaneously).
Heterozygote Comparison	Aa vs. AA	Evaluates whether carrying one minor allele (Aa) affects risk compared to the homozygous major allele (AA).
Homozygote Comparison	AA vs. aa	Assesses the effect of two copies of the major allele (AA) compared to two copies of the minor allele (aa).
Dominant	(Aa + aa) vs. AA	Tests the effect of carrying at least one minor allele (Aa + aa) against having only the major allele (AA)
Recessive	aa vs. (AA + Aa)	Evaluates if two minor alleles (aa) are necessary to observe an effect.
Over-dominant Model (Heterozygote Superiority)	Aa vs. (AA + aa)	Determines if heterozygotes (Aa) have an effect distinct from both homozygous genotypes (AA and aa).
Allelic/Multiplicative Model (Allelic Frequency)	a vs. A (or A vs. a)	Assesses the impact of each additional minor allele (a) compared to the major allele (A). “A versus a” indicates whether the major allele alters risk, while “a versus A” shows the effect of the minor allele.

A—major (common) allele, a—minor allele.

**Table 3:** Study population characteristics.

Parameter		Cases	Controls	*p*
Sample size		126	213	
Median age (range)		45.5 (25–79)	51 (18–87)	0.005
Menopausal status	Premenopausal (<51 y.o.)	77 (61.1%)	97 (45.5%)	0.006
	Postmenopausal (≥51 y.o.)	49 (38.9%)	116 (54.5%)	
Histology	Squamous	101 (80.2%)		
	Non-squamous	23 (18.3%)		
	N/a	2 (1.6%)		
Disease stage	Early	50 (39.7%)		
	Advanced	73 (57.9%)		
	N/a	3 (2.4%)		

Age: in years; N/a: Non-available for analysis (e.g., only grading available or stage missing).

**Table 4:** MAF values for the study cohort, *p*-values for Hardy–Weinberg equilibrium (HWE), and reference MAFs for European populations from gnomAD and/or the dbGaP ALFA project.

	MAF	HWE (*p* Value)	MAF in gnomAD/dbGaP ALFA
**IL-8**			
rs4073	A = 0.467	*p* = 0.78	A = 0.449 (gnomAD)/A = 0.454 (ALFA)
rs2227306	T = 0.437	*p* = 0.4	T = 0.42 (gnomAD)/T = 0.42 (ALFA)
rs2227543	T = 0.427	*p* = 1	T = 0.416 (gnomAD)/T = 0.415 (ALFA)
rs1126647	T = 0.411	*p* = 0.89	T = 0.41 (gnomAD)/T = 0.332 (ALFA)
**IL-16**			
rs11556218	G = 0.092	*p* = 0.14	G = 0.074 (gnomAD)/G = 0.079 (ALFA)
rs4778889	C = 0.136	*p* = 0.26	C = 0.175 (gnomAD)/C = 0.182 (ALFA)
rs4072111	T = 0.103	*p* = 0.59	T = 0.113 (gnomAD)/T = 0.107 (ALFA)
rs1131445	C = 0.324	*p* = 0.25	C = 0.347 (gnomAD)/C = 0.349 (ALFA)

MAF—minor allele frequency (in the present study), HWE—Hardy–Weinberg equilibrium, gnomAD—Genome Aggregation Database; ALFA—Allele Frequency Aggregator; dbSNP—Database of Single Nucleotide Polymorphisms; dbGaP—Database of Genotypes and Phenotypes.

**Table 5:** Genotype and allele frequencies of IL-8 SNPs among CC cases and healthy controls.

Model	Genotype	Controls	Cases	OR (95% CI)	*p* ^Fi^	χ^2^	*p* ^Chi^
**rs4073 (−251 A>T)**							
Co-dominant	TT	59 (27.7%)	35 (27.8%)			0.94 (df = 2)	0.626
Heterozygote	AT	109 (51.2%)	59 (46.8%)	0.91 (0.54–1.54)	0.789	0.12	0.729
Homozygote	AA	45 (21.1%)	32 (25.4%)	0.83 (0.45–1.55)	0.637	0.33	0.566
Dominant	AT + AA	154 (72.3%)	91 (72.2%)	0.996 (0.61–1.63)	1	0	0.988
AT + AA vs. TT	TT	59 (27.7%)	35 (27.8%)				
Recessive	AA	45 (21.1%)	32 (25.4%)	1.27 (0.76–2.14)	0.421	0.82	0.365
AA vs. TT + AT	TT + AT	168 (78.9%)	94 (74.6%)				
Overdominant	AT	109 (51.2%)	59 (46.8%)	0.84 (0.54–1.31)	0.5	0.6	0.439
AT vs. AA + TT	TT + AA	104 (48.8%)	67 (53.2%)				
Allele frequency	T	227 (53.3%)	129 (51.2%)	0.92 (0.67–1.26)	0.633	0.28	0.597
A vs. T	A	199 (46.7%)	123 (48.8%)				
**rs2227306 (+781 C>T)**							
Co-dominant	CC	64 (30%)	33 (26.2%)			0.58 (df = 2)	0.749
Heterozygote	CT	112 (52.6%)	70 (55.6%)	1.21 (0.72–2.03)	0.516	0.54	0.462
Homozygote	TT	37 (17.4%)	23 (18.3%)	0.83 (0.43–1.62)	0.610	0.3	0.584
Dominant	TT + CT	149 (70%)	93 (73.8%)	1.21 (0.74–1.98)	0.459	0.58	0.446
CT + TT vs. CC	CC	64 (30%)	33 (26.2%)				
Recessive	TT	37 (17.4%)	23 (18.3%)	1.06 (0.6–1.89)	0.883	0.04	0.841
TT vs. CT + CC	CT + CC	176 (82.6%)	103 (81.7%)				
Overdominant	CT	112 (52.6%)	70 (55.6%)	1.13 (0.72–1.76)	0.652	0.28	0.597
	TT + CC	101 (47.4%)	56 (44.4%)				
Allele frequency	C	240 (56.3%)	136 (54%)	1.10 (0.81–1.51)	0.576	0.36	0.549
T vs. C	T	186 (43.7%)	116 (46%)				
**rs2227543 (+1633 C>T)**							
Co-dominant	CC	70 (32.9%)	41 (32.5%)			0.03 (df = 2)	0.986
Heterozygote	CT	104 (48.8%)	61 (48.4%)	1 (0.61–1.65)	1	0	1
Homozygote	TT	39 (18.3%)	24 (19%)	0.95 (0.50–1.8)	1	0.02	0.887
Dominant	TT + CT	143 (67.6%)	85 (67.5%)	1.02 (0.63–1.62)	1	0	1
CT + TT vs. CC	CC	70 (32.9%)	41 (32.5%)				
Recessive	TT	39 (18.3%)	24 (19%)	1.05 (0.6–1.85)	0.886	0.03	0.862
TT vs. CC + CT	CT + CC	174 (81.7%)	102 (81%)				
Overdominant	CT	104 (48.8%)	61 (48.4%)	0.98 (0.63–1.53)	1	0.01	0.920
	CC + TT	109 (51.2%)	65 (51.6%)				
Allele frequency	C	244 (57.3%)	143 (56.7%)	0.98 (0.71–1.34)	0.936	0.02	0.888
T vs. C	T	182 (42.7%)	109 (43.3%)				
**rs1126647 (+2767 A>T)**							
Co-dominant	AA	73 (34.3%)	40 (31.8%)			0.46 (df = 2)	0.793
Heterozygote	AT	105 (49.3%)	62 (49.2%)	0.86 (0.47–1.58)	0.643	0.23	0.631
Homozygote	TT	35 (16.4%)	24 (19%)	1.25 (0.66–2.39)	0.511	0.46	0.498
Dominant	AT + TT	140 (65.7%)	86 (68.3%)	1.12 (0.7–1.79)	0.721	0.23	0.631
AT + TT vs. AA	AA	73 (34.3%)	40 (31.7%)				
Recessive	TT	35 (16.4%)	24 (19%)	1.2 (0.67–2.12)	0.556	0.38	0.538
TT vs. AT + AA	AT + AA	178 (83.6%)	102 (81%)				
Overdominant	AT	105 (49.3%)	62 (49.2%)	0.996 (0.64–1.55)	1	0	1
	AA + TT	108 (50.7%)	64 (50.8%)				
Allele frequency	A	251 (58.9%)	142 (56.3%)	0.9 (0.66–1.23)	0.521	0.43	0.512
T vs. A	T	175 (41.1%)	110 (43.7%)				

*p* ^Fi^—*p*-value in Fisher’s exact test, *p* ^Chi^—*p*-value in Chi-squared test (for df = 1 or df = 2).

**Table 6:** Genotype and allele distribution of IL-16 SNPs rs11556218, rs4778889, rs4072111, and rs1131445.

Model	Genotype	Controls	Cases	OR (95% CI)	*p* ^Fi^	χ^2^	*p* ^Chi^
**rs11556218 (T>G)**							
Co-dominant	TT	174 (81.7%)	103 (81.75%)	1.00 (Ref.)		N/a	N/a
Heterozygote	GT	39 (18.3%)	23 (18.25%)	0.996 (0.56–1.76)	1	0	1
Homozygote	GG	0 (0%)	0 (0%)	NaN	1	NaN	NaN
Dominant	GG + GT	39 (18.3%)	23 (18.25%)	0.996 (0.56–1.76)	1	0	1
(GG + GT vs. TT)	TT	174 (81.7%)	103 (81.75%)				
Recessive	GG	0 (0%)	0 (0%)	NaN	1	NaN	NaN
GG vs. GT + TT	GT + TT	213 (100%)	126 (100%)				
Overdominant	GT	39 (18.3%)	23 (18.25%)	0.996 (0.56–1.76)	1	0	1
(GT vs. TT + GG)	TT + GG	174 (81.7%)	103 (81.75%)				
Allele frequency	T	387 (90.8%)	229 (90.9%)	0.997 (0.58–1.71)	1	0	1
(G vs. T)	G	39 (9.2%)	23 (9.1%)				
**rs4778889 (T>C)**							
Co-dominant	TT	157 (73.7%)	86 (68.3%)	1.00 (Ref.)		2.68 (df = 2)	0.262
Heterozygote	CT	54 (25.4%)	40 (31.7%)	1.35 (0.83–2.2)	**0.259**	1.49	**0.222**
Homozygote	CC	2 (0.9%)	0 (0%)	∞ (NaN–∞)	0.542		
Dominant	CC + CT	56 (26.3%)	40 (31.7%)	1.3 (0.8–2.12)	**0.319**	1.16	**0.281**
CC + CT vs. TT	TT	157 (73.7%)	86 (68.3%)				
Recessive	CC	2 (0.9%)	0 (0%)	0 (0–∞)	0.532		
CC vs. CT + TT	CT + TT	211 (99.1%)	126 (100%)				
Overdominant	CT	54 (25.4%)	40 (31.7%)	1.37 (0.84–2.23)	**0.212**	1.62	**0.203**
CT vs. CC + TT	CC + TT	159 (74.6%)	86 (68.3%)				
Allele frequency	T	368 (86.4%)	212 (84.1%)	1.2 (0.77–1.85)	**0.430**	0.65	**0.420**
C vs. T	C	58 (13.6%)	40 (15.9%)				
**rs4072111 (C>T)**							
Co-dominant	CC	172 (80.8%)	93 (73.8%)	1.00 (Ref.)		2.32 (df = 2)	0.314
Heterozygote	CT	38 (17.8%)	30 (23.8%)	1.46 (0.85–2.51)	0.205	1.89	0.169
Homozygote	TT	3 (1.4%)	3 (2.4%)	0.54 (0.11–2.73)	0.669		
Dominant	TT + CT	41 (19.2%)	33 (26.2%)	1.49 (0.88–2.51)	0.173	2.24	0.134
TT + CT vs. CC	CC	172 (80.8%)	93 (73.8%)				
Recessive	TT	3 (1.4%)	3 (2.4%)	1.71 (0.34–8.59)	0.674	N/a	N/a
TT vs. CT + CC	CT + CC	210 (98.6%)	123 (97.6%)				
Overdominant	CT	38 (17.8%)	30 (23.8%)	1.44 (0.84–2.47)	0.207	1.76	0.185
CT vs. TT + CC	TT + CC	175 (82.2%)	96 (76.2%)				
Allele frequency	C	382 (89.7%)	216 (85.7%)	1.45 (0.90–2.32)	0.139	2.38	0.123
T vs. C	T	44 (10.3%)	36 (14.3%)				
**rs1131445 (T>C)**							
Co-dominant	TT	101 (47.4%)	55 (43.65%)	1.00 (Ref.)		0.465 (df = 2)	0.792
Heterozygote	TC	86 (40.4%)	54 (42.86%)	1.15 (0.72–1.85)	0.629	0.35	0.554
Homozygote	CC	26 (12.2%)	17 (13.49%)	0.83 (0.42–1.67)	0.720	0.27	0.603
Dominant	CC + TC	112 (52.6%)	71 (56.35%)	1.16 (0.75–1.81)	0.573	0.45	0.502
CC + TC vs. TT	TT	101 (47.4%)	55 (43.65%)				
Recessive	CC	26 (12.2%)	17 (13.5%)	1.12 (0.58–2.16)	0.738	0.12	0.729
CC vs. TC + TT	TC + TT	187 (87.8%)	109 (86.5%)				
Overdominant	TC	86 (40.4%)	54 (42.9%)	1.11 (0.71–1.73)	0.732	0.2	0.654
TC vs. TT + CC	TT + CC	127 (59.6%)	72 (57.1%)				
Allele frequency	T	288 (67.6%)	164 (65.1%)	1.12 (0.81–1.56)	0.555	0.45	0.502
C vs. T	C	138 (32.4%)	88 (34.9%)				

*p* ^Fi^—*p*-value in Fisher’s exact test, *p* ^Chi^—*p*-value in Chi-squared test (for df = 1 or df = 2). Significant *p*-values are in bold, NaN—not a number, N/a—not applicable.

**Table 7:** Genotype and allele distribution of IL-16 SNPs rs 4072111 and rs4778889 broken down by menopausal status.

Model	Genotype	Controls	Cases	OR (95% CI)	*p* ^Fi^	χ^2^	*p* ^Chi^
**rs 4072111 (C>T)**							
**Premenopausal**							
Co-dominant	CC	82 (84.5%)	54 (70.1%)	1.00 (Ref.)		6.55	**0.038**
Heterozygote	CT	15 (15.5%)	21 (27.3%)	2.13 (1.01–4.48)	0.059	4.02	**0.045**
Homozygote	TT	0 (0%)	2 (2.6%)			1	0.32
Dominant	TT + CT	15 (15.5%)	23 (29.9%)	2.33 (1.12–4.86)	**0.026**	5.22	**0.022**
TT + CT vs. CC	CC	82 (84.5%)	54 (70.1%)				
Recessive	TT	0 (0%)	2 (2.6%)	∞ (NaN–∞)		0.78	0.379
TT vs. CT + CC	CT + CC	97 (100%)	75 (97.4%)				
Overdominant	CT	15 (15.5%)	21 (27.3%)	2.05 (0.97–4.32)	0.062	3.65	0.056
CT vs. TT + CC	TT + CC	82 (84.5%)	56 (72.7%)				
Allele frequency	T	15 (7.7%)	25 (16.2%)	2.31 (1.17–4.56)	**0.017**	6.1	**0.014**
	C	179 (92.3%)	129 (83.8%)				
**Postmenopausal**							
Co-dominant	CC	90 (77.6%)	39 (79.6%)	1.00 (Ref.)		0.098	0.952
Heterozygote	CT	23 (19.8%)	9 (18.4%)	0.90 (0.38–2.13)	1	0.05	0.823
Homozygote	TT	3 (2.6%)	1 (2%)			<0.001	1
Dominant	TT + CT	26 (22.4%)	10 (20.4%)	0.89 (0.39–2.02)	0.839	0.08	0.777
TT + CT vs. CC	CC	90 (77.6%)	39 (79.6%)				
Recessive	TT	3 (2.6%)	1 (2%)	0.78 (0.08–7.74)	1	<0.001	1
TT vs. CT + CC	CT + CC	113 (97.4%)	48 (98%)				
Overdominant	CT	23 (19.8%)	9 (18.4%)	0.91 (0.39–2.14)	1	0.05	0.823
CT vs. TT + CC	TT + CC	93 (80.2%)	40 (81.6%)				
Allele frequency	T	29 (12.5%)	11 (11.2%)	0.89 (0.42–1.85)	0.854	0.11	0.740
T vs. C	C	203 (87.5%)	87 (88.8%)				
**rs 4778889 (T>C)**							
**Premenopausal**							
Co-dominant	TT	66 (68%)	54 (70.1%)	1.00 (Ref.)		NaN	
Heterozygote	CT	31 (32%)	23 (29.9%)	0.91 (0.47–1.73)	0.869	0.09	0.764
Homozygote	CC	0 (0%)	0 (0%)	NaN	1	NaN	NaN
Dominant	CC + CT	31 (32%)	23 (29.9%)	0.91 (0.47–1.73)	0.869	0.09	0.764
CC + CT vs. TT	TT	66 (68%)	54 (70.1%)				
Recessive	CC	0 (0%)	0 (0%)	NaN	1	NaN	NaN
CC vs. CT + TT	CT + TT	97 (100%)	77 (100%)				
Overdominant	CT	31 (32%)	23 (29.9%)	0.91 (0.47–1.73)	0.869	0.09	0.764
CT vs. CC + TT	CC + TT	66 (68%)	54 (70.1%)				
Allele frequency	C	31 (16%)	23 (14.9%)	0.92 (0.51–1.66)	0.881	0.07	0.791
C vs. T	T	163 (84%)	131 (85.1%)				
**Postmenopausal**							
Co-dominant	TT	91 (78.4%)	32 (65.3%)	1.00 (Ref.)		4.78	0.091
Heterozygote	CT	23 (19.8%)	17 (34.7%)	2.1 (1–4.42)	0.073	3.9	**0.048**
Homozygote	CC	2 (1.7%)	0 (0%)	0 (0–NaN)	1	<0.001	0.984
Dominant	CC + CT	25 (21.6%)	17 (34.7%)	1.93 (0.92–4.04)	0.082	3.14	0.077
CC + CT vs. TT	TT	91 (78.4%)	32 (65.3%)				
Recessive	CC	2 (1.7%)	0 (0%)	0 (0–NaN)	0.58	0.02	0.884
CC vs. CT + TT	CT + TT	114 (98.3%)	49 (100%)				
Overdominant	CT	23 (19.8%)	17 (34.7%)	2.15 (1.02–4.52)	0.049	4.15	**0.042**
CT vs. CC + TT	CC + TT	93 (80.2%)	32 (65.3%)				
Allele frequency	C	27 (11.6%)	17 (17.3%)	1.59 (0.82–3.08)	0.214	1.94	0.164
C vs. T	T	205 (88.4%)	81 (82.7%)				

*p* ^Fi^—*p*-value in Fisher’s exact test, *p* ^Chi^—*p*-value in Chi-squared test (for df = 1 or df = 2). Significant *p*-values are in bold, NaN—not a number.

## Data Availability

The data presented in this study are available on reasonable request from the first (R.W.) or the corresponding (E.O.) author.

## Appendix A

**Table A1:** Detailed clinicopathological characteristics of CC cases.

	n	Percent
**Histology**		
Squamous	101	80.2%
Adenocarcinoma	18	14.3%
Adenosquamous	5	4%
Missing	2	1.6%
**Grading**		
G1	10	7.9%
G2	53	42.1%
G3	40	31.8%
G-X	23	18.3%
**Pelvic lymph nodes**		
Positive	36	28.6%
Negative	72	57.1%
No PLND	18	14.3%
**Paraaortic lymph nodes**		
Positive	3	2.4%
Negative	85	67.5%
No PALND	38	30.2%
**FIGO (2018)**		
I A	12	9.5%
I B	36	28.6%
II A	2	1.6%
II B	25	19.8%
III A	5	4%
III B	6	4.8%
III C	32	25.4%
IV A	3	2.4%
IV B	2	1.6%
Missing	3	2.4%
**Treatment**		
Surgery alone	38	30.2%
Surgery followed by R(CH)T	22	17.5%
LN-staging followed by R(CH)T	48	38.1%
R(CH)T alone	3	2.4%
Neoadjuvant CHT followed by surgery	4	3.2%
RT followed by surgery	1	0.8%
CHT alone	1	0.8%
Missing	9	7.1%

PLND—pelvic lymphadenectomy, PALND—paraaortic lymphadenectomy, RT—radiotherapy, CHT—chemotherapy, R(CH)T—radiochemotherapy.
